# Refining fibroblast-to-cardiomyocyte transdifferentiation protocols to explore emergent self-organization in cardiac cultures

**DOI:** 10.1371/journal.pone.0343415

**Published:** 2026-03-23

**Authors:** Elena Turchaninova, Sofya Robustova, Sandaara Kovalenko, Vitalii Dzhabrailov, Aleria Dolgodvorova, Serafima Romanova, Dmitriy Zybin, Mikhail Popov, Alsu Miftakhova, Sheida Frolova, Mikhail Slotvitsky, Alexander Romanov, Konstantin Agladze, Valeriya Tsvelaya

**Affiliations:** 1 E. Meshalkin National Medical Research Center of the Ministry of Health of the Russian Federation, Novosibirsk, Russia; 2 Moscow Center for Advanced Studies, Moscow, Russia; 3 M.F. Vladimirsky Moscow Regional Clinical Research Institute, Moscow, Russia; 4 Novosibirsk State Medical University, Novosibirsk, Russia; Second Xiangya Hospital, CHINA

## Abstract

Fibrotic scars post-myocardial infarction disrupt cardiac conduction, causing arrhythmias. We developed a minimized 4-component cocktail (CHIR99021/BMP4/Activin A/IWP2) for efficient fibroblast-to-cardiomyocyte transdifferentiation. The use of the developed four-component protocol enables the generation of cells with electrical excitability and key cardiomyocyte markers, as evidenced by 56–83% of cells expressing α-actinin. This level of partial reprogramming of fibroblast cells into cardiac cells is sufficient to restore cardiac tissue conductivity, with an efficiency that exceeds the critical percolation threshold. Systemic delivery of components is safe, but requires further optimization, which will open up opportunities for localized delivery through smart substrates and combinations with cell therapy. Minimization of the transdifferentiation cocktail is not a compromise, but a strategic advantage that provides an optimal balance between functional efficiency and clinical applicability, including safety, delivery, and manufacturing.

## 1. Introduction

Cardiovascular diseases (CVDs) remain the principal cause of death globally. Myocardial infarction (MI), the acute and often fatal manifestation of ischemic heart disease (IHD), is a major driver of this burden. The American Heart Association’s Heart Disease and Stroke Statistics—2024 Update reports that in the United States alone, someone has a myocardial infarction approximately every 40 seconds [1]. Acute MI results from prolonged myocardial ischemia, triggering extensive cardiomyocyte necrosis. This initiates a maladaptive reparative response characterized by activation of resident cardiac fibroblasts (CFs), excessive deposition of extracellular matrix (ECM) [2], and the formation of a dense, non-contractile, and electrically non-conductive fibrotic scar [3]. This replacement fibrosis [2] disrupts the myocardial syncytium, severely impairing pump function and creating a substrate for lethal arrhythmias [4], directly contributing to heart failure and sudden cardiac death post-MI.

Novel therapeutic strategies for the post-MI fibrotic scar include pharmacological agents targeting neurohumoral activation and inflammation [5,6]; however, the primary solution remains surgical interventions, such as revascularization or device implantation [7]. While essential, these approaches fail to regenerate lost cardiomyocytes or restore physiological electrical conduction across the scar. Regenerative strategies, particularly cell replacement therapy using cardiomyocytes derived from induced pluripotent stem cells (iPSCs) [8] or mesenchymal stem cells (MSCs) [9], show promise for remuscularization [10]. However, they carry inherent risks of tumorigenicity [11] and arrhythmogenicity [12], alongside challenges of scalable cell production and immune rejection.

In situ direct reprogramming or transdifferentiation of resident CFs within the scar into induced cardiomyocyte-like cells (iCMs), bypassing pluripotency [13], offers a compelling alternative. CFs are a prime target due to their central role in post-MI scar formation and pathological remodeling [14] and their high abundance within the infarct zone [15]. Direct reprogramming circumvents the need for large-scale *in vitro* cell expansion (e.g., billions of cells per graft [10]) and avoids immune complications associated with allogeneic cell transplantation [16]. Multiple approaches exist for fibroblast to cardiomyocyte-like cells reprogramming, including viral delivery of transcription factors (e.g., Oct4, Sox2, Klf4 [17,18]; Gata4, Mef2c, Tbx5 (GMT) [19]; GHMT [20]), mRNA [21], and cocktails of small molecules. Small molecules modulate key signaling pathways (e.g., TGF-β, Wnt, cAMP) and include inhibitors of epigenetic repressors (tranylcypromine, valproate [22]), TGF-β inhibitors (RepSox, SB431542 [19,23,24]), Wnt modulators (CHIR99021 [22,24], XAV939 [21]), and cAMP activators (Forskolin [22,23]).

Despite progress, current transdifferentiation protocols, particularly the chemical-only protocol, suffer from critically low efficiency. Viral methods combined with small molecules enhance reprogramming *in vivo* [19,25,26] but face translational hurdles due to risks of insertional mutagenesis, immunogenicity, and manufacturing complexity [27]. Purely chemical cocktails offer superior safety and delivery flexibility (e.g., injectable, potentially oral [28]). However, reported conversion rates remain inadequate (e.g., ~ 1% after prolonged multi-dose regimens [28]), limiting functional recovery.

Crucially, complete reprogramming of all scar fibroblasts is likely unnecessary. One potential therapeutic strategy for post-MI scar treatment involves enhancing electrical conductivity to enable more synchronized contraction. This can be achieved by reprogramming a critical minimal fraction of fibroblasts into conductive iCMs, establishing interconnected conductive pathways that reach the electrophysiological percolation threshold within the scar tissue [29,30]. Achieving this threshold allows the electrical wavefront to propagate through the formerly inert scar [31].

This study directly addresses the challenge of low efficiency in chemical reprogramming for myocardial scar therapy, taking positive aspects of this fact. We hypothesize that a radically minimized, optimized chemical cocktail can achieve iCM conversion rates sufficient to reach the percolation threshold within the fibrotic scar. Minimization is of paramount importance for clinical translation as it increases the likelihood of the applicability of the cocktail. Complex cocktails pose significant challenges for pharmacokinetic profiling, safety assessment (including drug-drug interaction studies), formulation stability, manufacturing sequences, and regulatory approval pathways. Minimizing the number of compounds dramatically simplifies these processes, accelerating clinical development [32,33].

Additionally, we plan to use the chemical cocktails we have created for gradual targeted delivery to the myocardium. Implantable biomaterial scaffolds designed for localized, sustained, and temporally controlled release in the scar are a promising delivery platform. However, incorporating multiple compounds with potentially different release kinetics, stability requirements, and complex cross-linking and interactions is highly complex and often impractical. A minimal cocktail is necessary for the efficient development and performance of such next-generation delivery systems [34,35].

Such chemical cocktails also have the potential for synergistic combination therapies. A simple, effective, and minimal chemical reprogramming strategy could be easily combined with cell replacement therapies (e.g., iPSC-CM patches) to enhance their efficacy and overcome their differentiation-related drawbacks. Reprogramming a subset of scar fibroblasts in situ could create a more hospitable conductive microenvironment, improving survival, integration, and electromechanical coupling of grafted cardiomyocytes [36,37].

Therefore, we set out to develop, optimize, and validate a novel, minimal reprogramming chemical cocktail specifically designed to efficiently transdifferentiate fibroblasts to functional iCMs. Our primary goal was to achieve an efficiency sufficient to establish conductive pathways (above the percolation threshold) in models replicating the fibrotic environment following myocardial infarction, using both animal and human cells to demonstrate robust translational potential.

## 2. Results

### 2.1. In vitro transdifferentiation from fibroblast to cardiomyocyte-like cells (iCM)

The primary goal of this study section was to develop a small-molecule reprogramming protocol for a minimal set of factors that could drive a sufficient number of fibroblasts toward a conductive, cardiomyocyte-like fate to create new electrical pathways. By minimizing the number of active molecules, we reduce potential immunogenicity and manufacturing complexity, which is important for future clinical applications. As a baseline, we chose a protocol for controlling fibroblast-to-cardiomyocyte transdifferentiation [22] that was primarily used for mouse embryonic fibroblasts.

Starting from the base protocol [22], we focused on modulating the Wnt/β-Catenin signaling pathway, which plays a key role not only in directed differentiation [38] but also in transdifferentiation, according to [22,24]. From these latter two studies, we adopted the hypothesis and the variety of compounds modulating the core (Wnt/β-Catenin) and auxiliary pathways. Subsequently, we applied the logic for controlling the Wnt/β-Catenin dynamic system, as predicted by a computer Petri net model [39].

The model demonstrates that a threshold signal of GSK3 inhibition is required to switch the system’s state [39]. This function is performed by CHIR99021, and from these considerations, its initial concentration, similar to [22], was chosen. However, the model also shows that sustained activation inevitably triggers a powerful negative feedback loop via AXIN2, which counteracts the pathway stimulation [39]. A natural solution is to harness this feedback by adding IWP2 to our protocol to artificially and abruptly terminate Wnt/β-Catenin signaling (unlike [22]). This mimics the natural pathway attenuation and promotes stabilization of the new state. BMP4 and Activin A were added as necessary cooperative signals from the parallel cardiogenic network (Smad pathway). Their role is to lower the energy barrier for the transition between stable states (fibroblast → cardiomyocyte), providing synergy with the threshold activation of Wnt/β-Catenin. Moreover, IWP2 is introduced after the completion of cooperative induction to artificially quench the Wnt/β-Catenin, consistent with the model’s [39] prediction about the necessity of activating the negative feedback (AXIN2) to stabilize the new state. Therefore, the combination of literature data and the model [39] allowed us to define the minimal protocol core.

Performance was evaluated against two predefined success criteria: 1) α-actinin expression in >30% of cells, and 2) the formation of conduction clusters. A cocktail was deemed minimally sufficient if it met both criteria and the further removal of any component caused performance to fall below these thresholds. This process was conducted independently for rat and human fibroblasts, yielding the specific minimal combinations reported. The goal was to determine the minimum set of components to ensure the conditions for the formation of new conduction pathways, rather than to achieve full maturity or maximum efficiency of transdifferentiation.

This section describes the results of testing modified protocols for the transdifferentiation of fibroblasts into cardiomyocyte-like cells. The resulting cells were characterized, and the effectiveness of the protocol was assessed. For evaluation, several types of analysis were performed during transdifferentiation, including patch-clamp study, optical mapping of excitation wave propagation, and immunostaining for fibrillar actin, nuclei, and cardiomyocyte marker α-actinin.

#### 2.1.1. Rat embryonic fibroblasts derived iCM.

**Fig 1:**
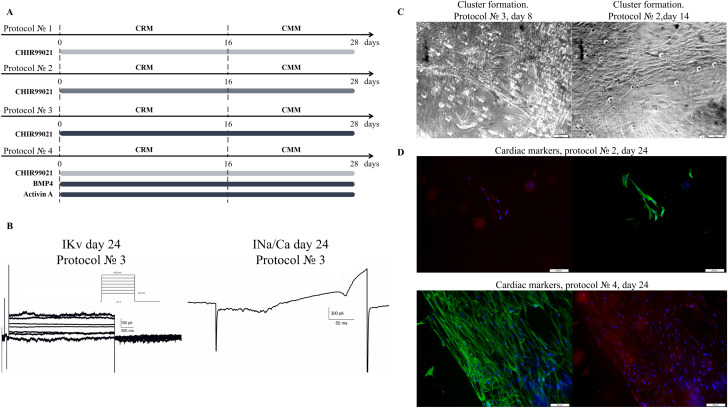
Results of rat embryonic fibroblasts (REF) direct reprogramming into cardiomyocyte-like cells. **(A)** The scheme of REF to cardiomyocyte-like cells direct reprogramming with small molecule CHIR99021 with varied concentrations 2.25, 4.5, 6.75 μM, marked by colour intensity and growth factors: Activin A 100 ng/mL and BMP-4 5 ng/mL. Fibroblasts were cultivated in the cardiac reprogramming medium (CRM) containing the small molecules and growth factors. On the 16th day, the medium was changed to cardiomyocyte maintaining medium (CMM). **(B)** Patch-clamp recordings of native voltage-gated ion currents in REF-derived cardiomyocyte-like cells. Representative whole-cell recordings from day 24 are shown. IKv — step protocol recording of delayed rectifier potassium current. INa/Ca — ramp protocol recording of composite voltage-gated sodium and calcium currents. **(C)** Morphology of REF-derived cell clusters on the 8th and 14th day of induction. The first clusters were observed on day 8-10. **(D)** Immunostaining for cardiac marker α-actinin (red), fibrillar actin (green), and nuclei (blue) in clusters generated from REFs on day 24. Scale bars 100 μm.

Firstly, we modified the transdifferentiation protocol for rat embryonic fibroblasts (REF) to iCM. The basic protocol was successfully adapted for REF by varying the concentrations of CHIR99021, Activin A, and BMP4 (Fig 1A), which were chosen as the main components. We compared four protocols for REFs with variable concentrations of small molecule CHIR99021 and tested the role of Activin A and BMP-4 supplementation for better cardiomyocyte induction. The final protocols for REF to cardiomyocyte-like cells direct reprogramming are presented in Fig 1A.

Cardiomyocyte-like cells used in this study were generated in four independent direct reprogramming experiments. All subsequent analyses for each protocol were conducted using cells from these biological replicates (n = 4). We observed changes in the cell’s morphology on day 8–10 (Fig 1C). Сlustering and strand formation indicate differentiation process success [22]. Electrophysiological profiling of cardiomyocyte-like cells on day 24 of transdifferentiation revealed the presence of composite INa/Ca and IKv currents. A representative whole-cell recording shows INa/Ca (recorded using a ramp protocol) with an amplitude of ∼150 pA and IKv with an amplitude of ∼200 pA (Fig 1B). Cells after 24 days of transdifferentiation were stained positively for cardiac markers. Some cells after transdifferentiation displayed partially striated α-actinin expression patterns (Fig 1D).

The percentage ratio of α-actinin expression to f-actin expression was measured on day 24: Protocol № 1–73 ± 10%, Protocol № 2–75 ± 3%, Protocol № 3–83 ± 8%, Protocol № 4–79 ± 21%. Particularly noteworthy is Protocol № 3, which resulted in a higher fraction of cells expressing the structural marker α-actinin content in cells compared to the other protocols, supposedly due to the high concentration of CHIR99021.

#### 2.2.2. Rat neonatal fibroblasts derived iCM.

**Fig 2:**
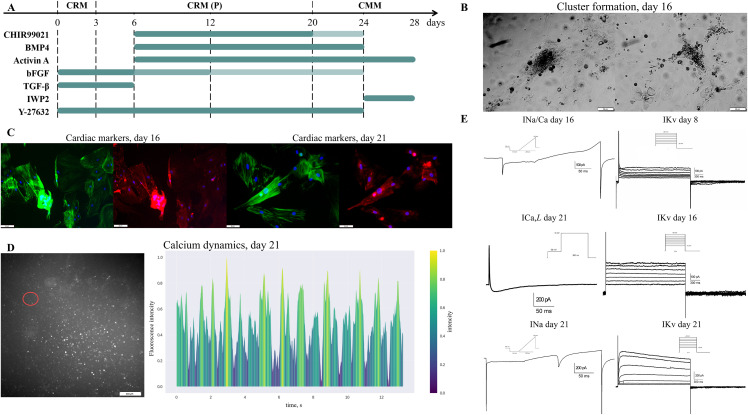
Results of direct reprogramming of rat neonatal fibroblasts (RNF) into cardiomyocyte-like cells. **(A)** The scheme of RNF to cardiomyocyte-like cells direct reprogramming. Fibroblasts were cultivated in a cardiac reprogramming medium (CRM) from day 3. CRM was replaced with CRM (P) from day 20, with CMM. Small molecule concentrations: CHIR99021 10.0, 3.0 μM marked by colour intensity, IWP2 2μM, Y-27632 5 μM; growth factors concentrations: BMP4 5.0 ng/ml, Activin A 9.0 ng/mL, bFGF 8.0, 5.0, 1.0 ng/ml marked by colour intensity, TGF-β 1.0 ng/ml. **(B)** Morphology of RNF-derived clusters induced by small molecules and growth factors. The first clusters were observed on day 12-16. **(C)** Immunostaining for cardiac marker α-actinin (red), fibrillar actin (green), and nuclei (blue) in clusters generated from RNFs on days 16 and 21. Scale bars 50 μm. **(D)** Excitation wave in RNF-derived cardiomyocyte-like cells on day 21. Left: selected region; right: normalized fluorescence intensity of calcium-dependent dye over time for selected region. Yellow colour indicates the times of peak calcium concentrations (this indicates the generation of excitation waves). **(E)** Patch-clamp recordings of voltage-gated ion currents in RNF-derived cardiomyocyte-like cells. Representative whole-cell recordings are shown. Left column: INa/Ca day 16 – ramp protocol recording of overlapping sodium and calcium currents; INa day 21 – ramp protocol recording of voltage-gated sodium current; ICa,L day 21 – step-protocol recording of L-type calcium current. Right column: IKv day 8, IKv day 16, IKv day 21 – representative current traces in response to depolarizing voltage steps (step-pulse protocol).

After testing protocols for REFs, we adapted them for neonatal rat fibroblasts (RNF) (Fig 2A). For survival enhancement, Y-27632, a selective ROCK inhibitor, was added to prevent apoptosis. Besides Activin A and BMP4 during early transdifferentiation stages, growth factor TGF-β, which activates the SMAD pathway, was applied. SMAD is believed to promote smooth muscle cell differentiation and apoptosis inhibition [40]. From the 24th day of transdifferentiation, analogous to human IPSC differentiation to cardiac tissue [41], the protocol was supplied with an IWP2 small molecule, an inhibitor of the Wnt pathway. Unlike the protocol for REF, during transdifferentiation of RNF (Fig 2A), CRM(P) medium combining CRM with 50% of 2–4 day medium from neonatal cardiomyocytes was used to exploit the paracrine effect [42].

The modified protocols for RNF to cardiomyocyte-like cells direct reprogramming are presented in Fig 2A. Cardiomyocyte-like cells used in this study were generated in four independent direct reprogramming experiments. All subsequent analyses were conducted using cells from these biological replicates (n = 4). Clusterization and strand formation were observed on day 12–16 of induction (Fig 2B).

**Fig 3:**
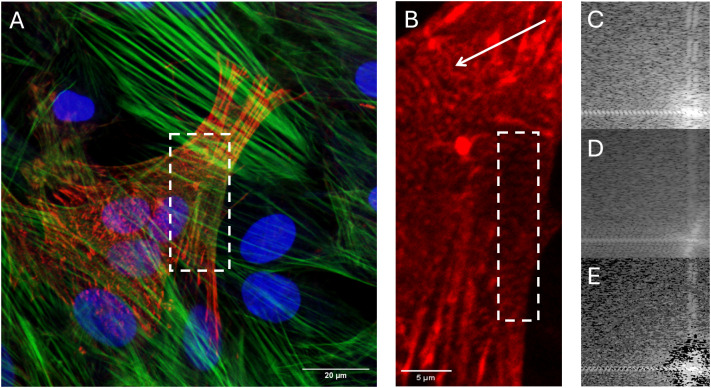
Cross-striations in RNF on day 16 of differentiation. **(A)** Immunostaining for the cardiac marker α-actinin (red), fibrillar actin (green), and nuclei (blue). The region of interest is indicated by a white rectangle. **(B)** Magnified view of the area with α-actinin cross-striations. An arrow points to one of the cross-striated zones in this cell. **(C, D)** Fourier transform of the area highlighted in panel b for: **(C)** – the α-actinin (red) channel and **(D)** – the fibrillar actin (green) channel. **(E)** Logical “AND” operation applied to the Fourier transforms from **(C)** and **(D)**.

RNFs were stained for cardiac marker α-actinin on different days of transdifferentiation; from the 16th day, a partially striated pattern of α-actinin expression was observed (Fig 2C,3). Fig 3C,3B show Fourier transforms of the α-actinin and fibrillar actin signals, respectively. As can be seen, the Fourier transformation reveals the clear presence of equally spaced bands in the confocal cell scan. To unambiguously determine the nature of the α-actinin bands (i.e., transverse cellular banding, rather than signal overlap from other cells or cytoskeletal features), a logical “AND” operation was performed on the Fourier spectra of the α-actinin and fibrillar actin signals (Fig 3E). Following this operation, it can be concluded that the bands of fibrillar actin and α-actinin are of different origins, as the area in Fig 3E indicating banding appears black (signifying a mismatch between the banding patterns in Fig 3C,3D).

During optical mapping with calcium-dependent dye Fluo-4 AM on the 21st day, we registered excitation propagation in response to electric pacing, confirming the successful induction into cardiomyocyte-like cells (Fig 2D). Qualitative patch-clamp recordings indicated progressive development of voltage-gated ion channels in RNF to cardiomyocyte-like cells (Fig 2E). Representative cells showed INa progression from day 16 (INa/Ca less than 100 pA) to day 21 (~200 pA), and IKv development from day 8 (~150 pA) through day 21 (~600 pA).

The results of the immunocytochemistry analysis showed an increase in the α-actinin/f-actin expression ratio from 62 ± 12% at day 16–77 ± 13% at day 21 of differentiation, indicating that the protocol effectively enhances α-actinin expression over time.

#### 2.2.3. Human atrial fibroblasts derived iCM.

For human atrial fibroblasts (HAF), primarily the RNF protocol was applied with slight modifications (Fig 3A). Instead of CRM(P), medium CRM(C) was applied for the enhancement of human fibroblast transdifferentiation.

**Fig 4:**
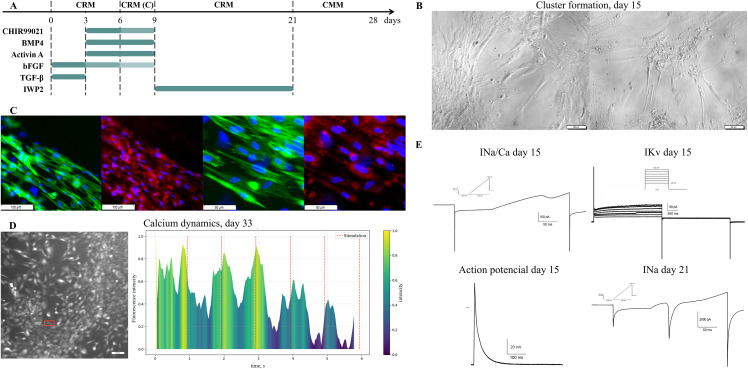
Results of direct reprogramming of human atrial fibroblasts (HAF) into cardiomyocyte-like cells. **(A)** The scheme of HAF to cardiomyocyte-like cells direct reprogramming with small molecules and growth factors. Fibroblasts were cultivated in CRM containing the small molecules and growth factors; on day 6, this was replaced with modified cardiomyocyte medium CRM (CRM(C)). On day 9, cells were returned to the CRM till day 21, when the medium was changed to CMM. Small molecule concentrations: CHIR99021 12.0, 10.0 μM marked by colour intensity, IWP2 2μM, growth factors concentrations: BMP4 5.0 ng/ml, Activin A 9.0 ng/mL, bFGF 8.0, 6.0, 4.0 ng/ml marked by colour intensity, TGF-β 1.0 ng/ml. **(B)** Morphology of HAF-derived clusters induced by small molecules and growth factors. The first clusters were observed on days 10-12. **(C)** Immunostaining for cardiac marker α-actinin (red), fibrillar actin (green), and nuclei (blue) in clusters generated from HAFs on day 33. Scale bars 100 μm (left) and 50 μm (right). **(D)** Excitation wave on day 33 of the induction. Left: selected region; right: normalized fluorescence intensity of a calcium-dependent dye over time for the selected region. Yellow colour indicates the times of peak calcium concentrations (this indicates the generation of excitation waves). The red dashed line marks the time of electrical pacing. **(E)** Patch-clamp recordings of voltage-gated ion currents and action potentials in HAF-derived cardiomyocyte-like cells. Representative whole-cell recordings are shown. Left column: INa/Ca day 15 — ramp protocol recording of overlapping voltage-gated sodium and voltage-gated calcium currents; Action potential day 15 — resting potential was about −80 mV. Right column: IKv day 15 — step protocol recording of summary potassium currents; INa day 21 — ramp protocol recording of voltage-gated sodium current.

The final optimized protocols for HAF to cardiomyocyte-like cells direct reprogramming are presented in Fig 4A. Cardiomyocyte-like cells used in this study were generated in four independent direct reprogramming experiments. All subsequent analyses were conducted using cells from these biological replicates (n = 4). We observed changes in cells’ morpholоogy on days 10–12. Clusterization and strand formation, large size, and single appearance may be features of successful differentiation (Fig 4B).

Optical mapping with calcium-dependent dye Fluo-4 AM was performed on the 21st and 33rd days. As can be seen, the excitation wave to electric pacing confirms the successful induction into cardiomyocyte-like cells (Fig 4D).

Patch-clamp analysis revealed the progressive development of voltage-gated ion channels (Fig 4E). On day 15, ramp protocols recorded composite INa/Ca currents (~115 pA), while depolarizing steps elicited small-amplitude potassium currents (IKv ≈ 110 ± 15 pA). Action potentials recorded the same day showed a normal resting membrane potential (~ −79 ± 3 mV) but lacked a plateau phase, resulting in a short duration (APD90: 42 ± 26 ms). By day 21, representative recordings showed substantially larger INa (~4000 pA), indicating robust sodium channel development. The emergence of nanoampere-range Na⁺ currents confirms progressive electrophysiological maturation and the protocol’s capability to generate cells with robust excitability. However, consistent gigaseal formation proved challenging at later stages, precluding reliable quantification of Ca²⁺ and delayed rectifier K⁺ currents.

Consistent with the electrophysiological study, immunofluorescent analysis on day 33 confirmed that HAFa were positive for cardiomyocyte marker α-actinin, and these cells also displayed partially striated α-actinin expression patterns (Fig 4C). Additionally, the α-actinin to f-actin expression ratio in these cells at day 33 of differentiation was measured at 56 ± 24%.

### 2.3. Testing transdifferentiation cocktail *for in vivo* application

#### 2.3.1. Safety testing for *in vivo* application of transdifferentiation cocktail.

**Fig 5:**
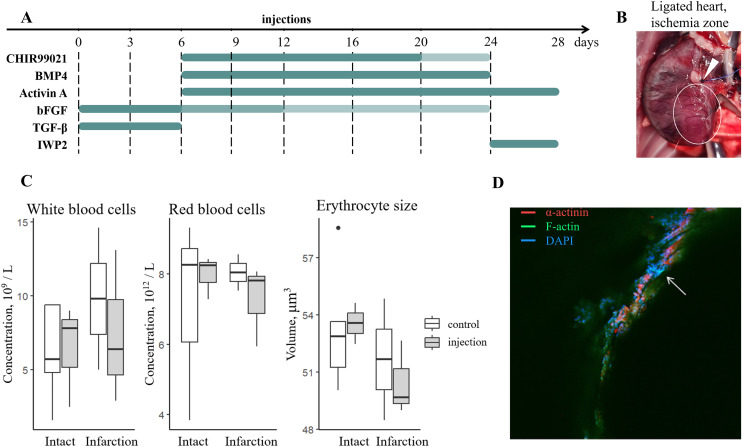
Rat infarction model for *in vivo* transdifferentiation. Safety testing. **(A)** The scheme of *in vivo* transdifferentiation in a rat infarction model. Small molecule concentrations: CHIR99021 10.0, 3.0 μM marked by colour intensity, IWP2 2μM; growth factor concentrations: BMP4 5.0 ng/ml, Activin A 9.0 ng/mL, bFGF 8.0, 5.0, 1.0 ng/ml marked by colour intensity, TGF-β 1.0 ng/ml. **(B)** Rat heart 30 minutes after ligation, ligation site shown with arrow, ischemic zone highlighted in white. **(C)** Clinical blood analysis data grouped by treatment type. Control in the intact group refers to a single PBS injection in the infarction group for non-treated animals. Treatment consists of reprogramming formulation injection: in the intact group – a single intramyocardial injection, in the group with infarction – repeated intravenous injections according to the protocol. White blood cell concentration, red blood cell concentration, and red blood cell size are illustrated on boxplots; no statistically significant difference was observed. **(D)** Immunostaining of cardiac marker α-actinin (red) in the damaged zone 1 month after infarction. Green color is f-actin staining. Nuclei were stained with DAPI (blue). The white arrow shows the damaged zone. The scale bar represents 200 μm.

For *in vivo* testing, the protocol for RNF, excluding ROCK inhibitor Y-27632, was adopted (Fig 5A). Since the duration of reprogramming is important for cell maturation, and *in vitro* takes at least 3–4 weeks [22,24], we performed repeated injections of components for induction with almost the same intervals as in *in vitro* protocol for RNFs (Fig 2A) i.e. no more than 2 injections a week, that was found safe for control protocol administration in mice [28]. Treatment started 14 days according to the scheme (Fig 5A) after myocardial infarction induction via coronary artery ligation (Fig 5B). Fig 5D presents an example of immunohistochemical characterization of heart fibrosis in our infarction model.

Firstly, we have checked the safety issues with the *in vivo* application of transdifferentiation in a heart attack treatment experiment. Toxicity tests were performed on intact animals administered a single dose of intramyocardial injection. No side effects were observed during the three-day observation period. Clinical blood analysis also showed no significant difference in leukocyte formula and hemoglobin-related parameters between treated and non-treated animals (Fig 5C).

Since intramyocardial injections can only be performed under general anesthesia, repeated interventions of this kind are not safe for animals. Thus, during transdifferentiation *in vivo* testing, we performed intravenous injections as a compromise between targeting and safety.

#### 2.3.2. Illustration of the efficiency of *in vivo* transdifferentiation in a rat infarction model.

**Fig 6:**
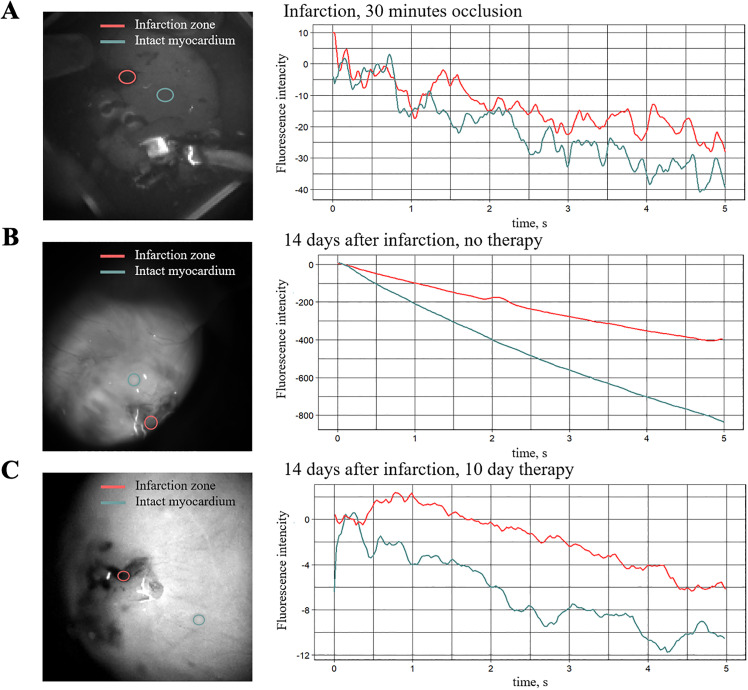
Characterization of the infarction zone by NADH fluorescence. Left — heart image with selected regions of infarction zone (red) and intact myocardium (green); right — Decrease of NADH autofluorescence intensity in the regions plotted in relative scale. **(A)** Infarction after 30 minutes of occlusion. **(B)** Infarction without treatment on the 14th day after surgery. **(C)** Infarction with a 10-day course of treatment, the treatment started on the 14th day after surgery.

We compared NADH fluorescence, a marker of ischemia and cellular respiration, in the rat heart during an *ex vivo* experiment. As is known, the process of NADH fluorescence intensity reduction is associated not only with its photobleaching but also with the activity of glutamate dehydrogenase, which regenerates NADH, thereby slowing the decline in fluorescence intensity [43]. Thus, by comparing the dynamics of NADH photobleaching, we can perform a qualitative assessment of tissues (Fig 6).

We compared the decrease in NADH fluorescence intensity in two regions of a Langendorff-perfused rat heart with myocardial infarction: the infarcted area and the intact area. The closer the NADH photobleaching curves are to each other, the more similar the infarct scar can be considered to healthy tissue. In Fig 6A, the curves are quite similar, which is due to the fact that the infarction in this heart was induced just 30 minutes before recording the curves, so the differences between the infarcted and intact regions are minimal.

In contrast, Fig 6B shows a significant difference in fluorescence decay between the compared regions, indicating that after 14 days of occlusion, the infarcted area undergoes substantial fibrotic changes. However, with therapy (Fig 6C), the photobleaching curves become similar again, suggesting that the treatment has a significant effect on the infarcted region of the heart, bringing the cellular respiration characteristics of the fibrotic area closer to those of healthy cardiomyocytes.

**Fig 7:**
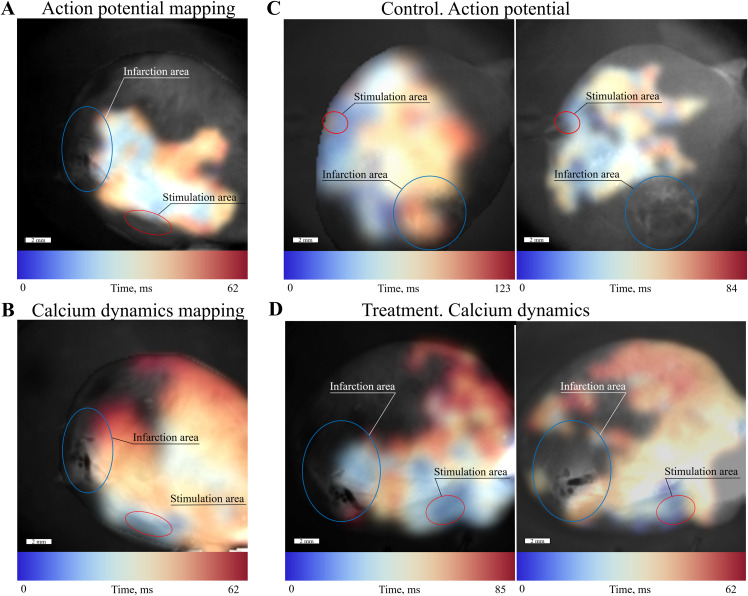
Characterization of chronic myocardial infarction in rat hearts on activation maps. The infarction zone is marked by a blue circle, and the stimulation area is indicated by a red circle. A color-coded time scale shows the spatial resolution of wave propagation. **(A)** Action potential propagation (Di-8-ANEPPS staining). **(B)** Calcium wave propagation (Fluo-4 AM staining) in the same infarcted heart. **(C)** Action potential propagation in the infarcted heart without treatment. **(D)** Calcium wave propagation in the infarcted heart following therapy.

To characterize functional improvement during therapy, we also adapted optical mapping methodology to assess excitation propagation in whole hearts during Langendorff perfusion. We observed certain differences in conduction in the infarction and intact zones of hearts (Fig 7). We used BDM in the perfusion solution to suppress cardiac muscle contraction. Therefore, the interpretation is focused on conduction patterns (i.e., the propagation of calcium and action potential waves) rather than contractility patterns. During experiment two dyes were applied: action potential sensitive Di-8-ANEPPS (Fig 7A) and calcium sensitive Fluo-4 AM (Fig 7B) — displayed comparable pattern on the same heart, and since the action potential wave and the subsequent calcium wave in the absence of drugs affecting their coupling have the same distribution patterns [44], they can be compared with each other.

In the two experimental groups: control (Fig 7C) and therapy (Fig 7D), the ratio of the conductive area to the total mapped heart area was measured. The results of this rough estimation demonstrate that the transdifferentiation treatment has a somewhat positive effect on cardiac conduction, increasing the proportion of conductive tissue from 71 ± 2% in the control group to 84 ± 3% in the treated group. This increase in the conductive ratio might indicate a relative reduction in the infarction area, suggesting partial restoration of conductivity in fibrotic cardiac tissue following therapy compared to untreated conditions.

## 3. Discussion

Our study demonstrates that a radically minimized chemical cocktail can successfully drive fibroblast to cardiomyocyte-like cells transdifferentiation (TdCM) in rodent and human models, reaching functional maturity sufficient to establish conduction pathways. We have previously demonstrated that the threshold of conduction cells in cultured cardiomyocytes and in cardiac tissue sufficient for conduction can be around 20–30%, which is the percolation threshold in the myocardium [45,46]. It is important to emphasize that this threshold, established in simplified *in vitro* models (monolayers) and theoretical calculations, is model-dependent and serves as an important mechanistic rationale, but requires further validation under complex *in vivo* conditions. Importantly, we show that partial reprogramming (~56–83% α-actinin cells⁺) can achieve the electrophysiological percolation threshold in fibrotic tissue. We demonstrated electrical propagation without the need for complete cellular conversion of fibroblasts. This is consistent with our central hypothesis of minimizing the cocktail composition for use in therapies or smart scaffolds.

This study demonstrates that a radically minimized chemical cocktail can successfully reprogram fibroblasts of different species (REF, RNF, HAF) into functionally competent iCMs. Crucially, the resulting iCMs exhibited key functional properties, especially, electrophysiological maturation progressed from early IKv currents (~100 pA at day 8) to robust INa (~4000 pA in HAFs by day 21), confirming excitability development (Figs 2E, 4E). Action potential generation in HAFs at day 15 (resting potential: −80 mV, APD90: ~ 42 ± 26ms) (Fig 4E), despite the absence of a plateau phase—a known hallmark of immaturity linked to CACNB2 splicing defects [47,48]. The excitation wave responded to electrical pacing (Figs 2D, 4D), confirming excitation-contraction coupling.

Reduced number of components (compared to conventional protocols [22,24]) enables feasible integration into implantable biomaterials for localized, sustained release. Complex cocktails with more than five components face insurmountable challenges in release kinetics, stability, and intra-scaffold cross-talk [49]. Our 4-component core (CHIR99021, BMP4, Activin A, IWP2) optimized physicochemical properties for encapsulation in hydrogel systems (e.g., alginate [49]), facilitating gradual paracrine delivery directly to the scar—a paradigm shift from systemic administration. Furthermore, minimalist cocktails allow for hybrid approaches where reprogrammed fibroblasts provide as a conductive “bridge” for iPSC-derived cardiomyocyte grafts (iPSC-CMs). *In vitro* induced CMs exhibited normal resting potentials (−80 mV) and progressive maturation of ion channels (INa ~ 4000 pA), confirming their ability to support electromechanical coupling. This addresses a key limitation of autonomous cell therapy: poor graft-host integration due to fibrotic isolation [25,50].

We focused on these four components when modifying the protocol because they are the key inhibitors and activators of signaling pathways in the process of direct differentiation into cardiomyocytes from mesenchymal stem cells and iPSCs [51]. CHIR99021 inhibits GSK3 protein kinase, promoting the proliferation of cardiomyocytes [52]. Activin A and BMP4, from the TGFβ superfamily, activate SMAD signaling, which regulates pathological processes [53]. Activin A also promotes bFGF expression [54], enhancing cell self-renewal. BMP4 prevents neurogenic differentiation and induces cardiac mesoderm formation in mice [55].

The reduction of components simplifies pharmacokinetic profiling, safety testing (e.g., drug interactions), and GMP-compliant manufacturing [56]. Our *in vivo* safety data (no hematological toxicity; Fig 5C) highlight the advantage of biocompatibility compared to viral methods or multidrug regimens [27].

While conversion efficiency (56–83% α-actinin⁺ cells) was modest compared to complex protocols [22,24], our approach prioritizes clinical translation over maximal cellular reprogramming. *In vivo* optical mapping revealed 84% conductive area in treated infarcts (compared to 71% in controls; Fig 7D), confirming that sparse iCM networks can re-establish functional electrical connectivity, effectively bridging the scar via percolation [57]. NADH fluorescence further demonstrated metabolic recovery in scars (Fig 6C), reflecting the physiological ability to restore conductance. This supports our paradigm: efficacy depends on achieving a critical conductance density rather than maximal reprogramming. The *in vivo* conductivity data, including the area of the conductive zone and the absence of significant arrhythmias in our experiments, provide important preliminary proof-of-concept for partial reprogramming. However, these results should be interpreted as supportive but still insufficient to precisely define a clinically relevant minimal effective fraction of reprogrammed cells. The present study expands the theoretical percolation model into a living system, representing a significant advance. A critical question for translation is the longer-term stability of this minimally induced phenotype. While our data show functional integration at the studied time points, it remains to be determined whether the electrophysiological improvements persist, diminish, or even progress towards greater maturity over extended periods. Investigating the potential for functional loss or phenotypic reversion is essential to strengthen the translational outlook for this minimal cocktail concept.

The electrophysiological characterization in this study was primarily focused on corroborating key functional insights derived from optical mapping, rather than providing an exhaustive quantitative description of ionic currents. Specifically, patch-clamp analysis was employed to establish the mechanistic basis of the calcium transients observed during mapping. Critically, this approach confirmed that the observed activity is triggered by transmembrane depolarizing currents (such as the sodium current, INa), rather than arising from purely intracellular calcium cycling independent of membrane depolarization. This confirmation is fundamental for demonstrating genuine electromechanical coupling and the functionality of the induced cardiomyocytes (iCMs).

The obtained patch-clamp recordings, albeit limited in number due to the technical challenges associated with heterogeneous cultures, qualitatively demonstrated the presence of depolarizing currents capable of initiating action potentials in the reprogrammed cells. The progressive development of nanoampere-range INa in human fibroblast-derived cells by day 21 provides direct evidence for the establishment of an excitable membrane. As expected, the amplitudes of recorded currents (INa, ICa,L, and IKv) in rat-derived iCMs were lower than those reported for fully mature cardiomyocytes, consistent with a partially reprogrammed, developing phenotype at this stage of maturation.

Therefore, the combination of methodologies—where optical mapping revealed propagating excitation waves and targeted patch-clamp analysis confirmed their transmembrane origin—provides sufficient evidence for the functional maturation of the generated iCMs within the scope of our objective: achieving the percolation threshold necessary to restore electrical conduction across scar tissue. For future work aimed at detailed quantitative profiling of ionic currents, increasing sample sizes are planned.

This study has some notable limitations due to its focus. For example, the lack of AP plateau in HAF-iCMs (Fig 4E) reflects underexpression of Cav1.2 [58]. In future studies, we may supplement thyroid hormone to scaffolds to improve maturation [34]. At this stage of the study, we were searching for a possible cocktail to integrate into the targeted delivery system. Of course, intravenous injections (used here for safety testing) limit specific targeting to the scar. It should be emphasized that currently published studies presenting results of *in vivo* fibroblast-to-cardiomyocyte direct reprogramming using small molecules also utilize systemic repeated delivery [19; 28], in contrast to direct reprogramming by retroviral transduction, which is performed via multiple intramyocardial injections right after infarction modeling [25,26].

In the future, we plan to develop smart scaffolds for localized release and combine the resulting cocktail with cell therapy, as in our previous study. That is, a combination therapy for co-delivery of the cocktail with iPSC-CMs on bifunctional scaffolds is possible [59]. The next critical step will be to rigorously test chronic efficacy and long-term stability in animal models of myocardial infarction. This planned study will employ scaffold-based delivery of the minimal cocktail in a chronic infarction model. Key readouts will include: 1) Long-term electrophysiological stability: serial optical mapping to monitor the persistence and evolution (e.g., conduction velocity, arrhythmia inducibility) of the conductive network; 2) Phenotypic durability: histological and molecular analysis (e.g., α-actinin, Cx43, ion channel expression) of the reprogrammed area to assess cell survival and stability of the cardiomyocyte-like phenotype; 3) Structural and metabolic remodeling: analysis of scar size, fibrosis content, and metabolic markers (e.g., NADH fluorescence). In this work, we conducted about six pilot experiments in rats to demonstrate the effect of the selected cocktails; however, this is not the major conclusion.

Thus, by focusing on cocktail minimization, we achieve sufficient electrophysiological activity and iCM maturity to partially restore scar conductance at the percolation threshold, as confirmed in human and weight-bearing cells. This strategy bridges the gap between regenerative potential and clinical feasibility by enabling the next translational step using scaffold-based delivery.

## 4. Materials and methods

### 4.1. Laboratory animals and ethical approval

All procedures were carried out in compliance with the National Institutes of Health Guide for the Care and Use of Laboratory Animals and were conducted in accordance with the Declaration of Helsinki. The study was approved by the Ethics Committee of M.F. Vladimirsky Moscow Regional Clinical Research Institute (protocol № 7 from 18.04.2024) and by the Moscow Institute of Physics and Technology Life Science Center Provisional Animal Care and Research Procedures Committee (Protocol No. A2-2012-09-02). All participants provided written informed consent using the form approved by the Ethics Committee. Only adults over 18 years of age were included in the study.

A total of 29 rats participated in the experiments, 6 of them – for perfusion. The success rate of all operations was more than 96%; only one rat died during the operation. All rats were anesthetized using the RWD R500 Small Animal Anesthesia Machine using oxygen and isoflurane. No rats were anesthetized more than once a day. Animals were randomly assigned to treatment or control groups. This pilot study was designed with the following predefined endpoints to assess safety and gather preliminary functional data:

1. Primary (Safety) Endpoints: Systemic toxicity evaluated via hematological and serum biochemical analysis in all animals.
2. Secondary (Exploratory) Endpoints: In this pilot study, preliminary efficacy was assessed as a proof of concept in the infarct model groups by quantifying the area of conductive tissue within the scar using optical mapping.

### 4.2. Rat embryonic fibroblasts cell culture

Rat embryonic fibroblasts were isolated from embryonic day 12–14 rat embryos [60]. For the embryos, the head and liver were carefully removed. The remaining tissues were placed in a trypsin solution until a single-cell suspension was obtained. Rat embryonic fibroblasts were cultured in fibroblast growth medium consisting of Dulbecco’s Modified Eagle Medium/Nutrient Mixture F-12 (DMEM/F-12, Gibco, 11320033), 15% Serum Replacement (SR, Gibco, 10828028), 1% Glutamax (Gibco, 35050061), 1% non-essential amino acids (NEAA, Gibco, 11140050), 100 units/ml penicillin and 100 μg/ml streptomycin (Paneco, А065п), 0.1 mM 2-Mercaptoethanol (Amresco), 10 ng/ml FGF-2 (Paneco, ФР-07050).

### 4.3. Rat neonatal fibroblasts cell culture

Rat neonatal fibroblasts were isolated using a two-day isolation protocol from Worthington-Biochem for rat neonatal cardiomyocytes and fibroblasts (http://www.worthingtonbiochem.com/NCIS/default.html). In brief, hearts were extracted from rat pups (Rattus norvegicus, Sprague Dawley breed), aged 1–4 days, and immediately placed in Ca2 + - and Mg2 + -free Hank’s Balanced Salt Solution (HBSS, Gibco, 14170112) on ice. Only the tissue of the ventricles was isolated—with approximately 50–60% of the initial heart mass being cut off—which included the sinoatrial node, the atria, and the atrioventricular node. The isolated ventricles were minced into small pieces and then left at 4 °C overnight for trypsinization (Trypsin-EDTA 0.25%, Gibco, 25200056). On the second day, the cells were placed into a collagenase solution (Collagenase type II, 2.25 μg/mL, Gibco, 17101015) and stirred for 1 hour at 37 °C. Next, the cells in suspension were placed into a T75 flask for 1 hour for pre-plating. The cells remaining in the flask after pre-plating were considered fibroblasts and were used with purification from cardiomyocytes.

### 4.4. Human atrial fibroblasts cell culture

Human atrial fibroblasts were isolated from atrial appendages provided by surgeons. The appendages were immediately placed in the cardioplegic solution Custodiol after excision and transported to the laboratory on ice. Fibroblast growth medium consists of Dulbecco’s Modified Eagle Medium (DMEM, Paneco, С410п), 10% Fetal Bovine Serum (FBS, Capricorn, FBS-HI-12B), 100 units/ml penicillin and 100 μg/ml streptomycin (Paneco, А065п). An atrial appendage was cut into pieces and then dispersed on gelatin-coated (Paneco, Ф061) 10-cm culture dish containing 2 mL of fibroblast growth medium; an additional 8 mL of medium was supplemented after 2 hours. Our extraction method is based on a well-known protocol [61].

### 4.5. Transdifferentiation protocols

As a starting point, we chose the protocol for controlling the transdifferentiation of fibroblasts into cardiomyocytes [22]. All abbreviations and main steps are consistent with this protocol. CRM (cardiac reprogramming medium) consisting of KnockOut DMEM (Gibco, 10829018), 15% Fetal Bovine Serum (FBS, Capricorn, FBS-HI-12B), 5% Serum Replacement (SR, Gibco, 10828028), 0.5% N2 supplement (Paneco, ФР-0205), 2% B27 supplement (Gibco, 17504044), 1% Glutamax supplement (Gibco, 35050061), 1% non-essential amino acids (NEAA, Gibco, 11140050), 0.1 μM 2-Mercaptoethanol (Amresco), 100 U/ml penicillin and 100 mg/ml streptomycin (Paneco, А065п) and 50 mg/ml vitamin C (Sigma-Aldrich, A4403). CMM (medium for maintenance of cells differentiated into cardiomyocytes) was prepared based on Dulbecco’s Modified Eagle Medium (DMEM, Paneco, С410п) supplemented with 15% Fetal Bovine Serum (FBS, Capricorn, FBS-HI-12B), 50 mg/ml vitamin C (Sigma-Aldrich, A4403), 3 μM CHIR99021 (Sigma-Aldrich, SML1046) and 1 mg/ml insulin (Paneco, Ф062). Medium from neonatal cardiomyocytes: Dulbecco’s Modified Eagle Medium (DMEM, Paneco, С410п) supplemented with 10% Fetal Bovine Serum (FBS, Capricorn, FBS-HI-12B), described above, after 2–3 days of culturing cardiomyocytes. CRM (P) refers to the combination of 50% CRM with 50% of medium from neonatal cardiomyocytes; аll additives’ concentrations refer to the total medium volume. CRM (C) refers to a cultural medium combining 50% of fresh CRM and 50% of 3-day CRM from the same well of the plate; аll additive concentrations refer to the total medium volume.

Fibroblasts’ transdifferentiation was started when fibroblasts reached 80% confluence in 24-well plates. On day 0 of each protocol, culture medium was changed to CRM with reprogramming factors. Medium was changed every 2–3 days with the addition of reprogramming factors. On the 16th or the 20th day, CRM was replaced with CMM.

The main components added to the environments were two small molecules: CHIR99021 stock solution 36 mM in DMSO, and IWP2 stock solution 5 mM in DMSO. Also growth factors was added: Activin A stock solution 50 μg/ml in 0.1% BSA (Sigma-Aldrich), BMP4 stock solution 10 μg/ml in 0.1% BSA (Sigma-Aldrich), TGF-β stock solution 10 μg/ml in 0.1% BSA (Sigma-Aldrich), FGF2 stock solution 10 μg/ml, Y-27632 (ROCK inhibitor) stock solution 5 mM in Phosphate Buffered Saline (PBS, Gibco, 10010023).

### 4.6. Immunofluorescent staining

Cell fixation occurred after study by optical mapping depending on the transdifferentiation protocol. Cells were fixed for 10 minutes in 4% paraformaldehyde (Sigma-Aldrich) and permeabilized for 10 minutes in 0.4% Triton-X100. Cells were further incubated for 1 hour in a blocking buffer (1% Bovine Serum Albumin (BSA, Sigma-Aldrich) in Phosphate Buffered Saline (PBS, Gibco, 10010023)), overnight at 4 °C with primary antibodies, and for 1 hour at room temperature with secondary antibodies. Cells were washed twice in PBS. Nuclei and F-actin were stained with DAPI (Vector Laboratories) and Alexa Fluor™ 488 Phalloidin (Thermo Fisher Scientific). Samples were analyzed and processed on a Zeiss LSM 710 confocal microscope with Zen black 3.0 software (Carl Zeiss AG). Primary antibodies (working dilutions—1:100): sarcomeric α-actinin mouse (Abcam, Cambridge, UK, Cat# ab9465). Secondary antibodies (Thermo Fisher Scientific, Waltham, MA, USA, working dilution—1:400): Alexa Fluor 594 goat anti-mouse IgG (HþL) highly cross adsorbed (A11005).

### 4.7. Patch-сlamp

Voltage-gated ion channel currents in transdifferentiated cardiomyocytes were recorded using the patch-clamp electrophysiological method in the “perforated whole-cell” configuration. A study using the patch-clamp method is described in another article [62]. The extracellular solution for recording sodium (INa), calcium (ICa,L), and potassium (IKv) contained 15 mM HEPES, 150 mM NaCl, 5.4 mM КCl, 1 mM MgCl2, 1.8 mM CaCl2, 15 mM D-glucose, pH 7.4 (adjusted with NaOH). The pipette was filled with an intracellular solution containing 150 mM KCl, 5 mM NaCl, 2 mM CaCl2, 5 mM MgATP, 10 mM HEPES, 5 mM EGTA, pH = 7.2 (adjusted with KOH). Currents from voltage-gated fast sodium channels (INa) as well as combined INa/Ca were obtained via the ramp stimulation protocol, which involved a linear increase in potential from –120 to +50 mV over a duration of 250 ms. Using a step-pulse stimulation protocol, current amplitudes of voltage-gated potassium channels (IKv) were quantified during depolarizing voltage steps (from −30–60 mV, each step presented for 2.5 s). The step protocol, which contains a pre-step (−30 mV) and main step (0 mV for 300 ms), was used to observe L-type calcium currents (ICa,L). The action potential (AP) was recorded using a 1 nA current stimulus with a duration of 2.5 ms. All experiments were conducted at room temperature (22–24°C). The membrane capacity ranged from 15 to 35 pF. Recordings were obtained from a limited number of cells per condition. Patch-clamp recordings were presented as qualitative indicators of ion channel development. Data analysis and processing were performed using Clampfit 10.2 (Molecular Devices, USA) and OriginPro 8.1 (OriginLab Corp., USA) software.

### 4.8. Infarction model

Male Wistar rats weighing 250−350 g were anesthetized with an injection combining “Xyla” xylazine hydrochloride 5 ng per 1 g of animal weight (De Adelaar, Netherlands) and “Zoletil” 4 ng tiletamine hydrochloride and 4 ng zolazepam hydrochloride per 1 g of animal weight (Vibrac, France). The trachea was intubated with an 18G intravenous cannula and connected to an Ambu bag (1 squeeze per second) and the RWD R550 Small Animal Anesthesia Machine, which combines 0.5–0.7 L/min oxygen with isoflurane via tubes. Left anterior descending coronary artery permanent occlusion was performed with monofilament Prolene 6/0 [63]. At the end of the surgery, rats were peritoneally administered meloxicam 2 μg/g of animal weight (Belkarolin, Belarus). The animal was monitored for several hours until it regained consciousness.

### 4.9. Intracardiac injections

Injections were performed intramyocardially or intravenously using a 30G insulin syringe with a total volume of 50–100 μl. Intramyocardial injections were performed only on control rats under general isoflurane anesthesia, no more than once with cocktail: CHIR99021–10 μM, Activin A — 9 ng/ml,BMP 4–5 ng/ml, IWP2–2 μM, bFGF — 8 ng/ml, TGFβ — 1 ng/ml per 20 ml of circulating blood volume — or with sterile Phosphate Buffered Saline (PBS, Gibco, 10010023). Intravenous injections into the tail vein for IM model animals were started on the 14th day after the surgery and were performed for 28 days, with no more than 2 injections per week.

### 4.10. Perfusion heart experimental protocol

The Langendorff perfusion protocol and perfusion scheme are based on previously published protocols [64]. The protocol for isolating the heart and cannulating the aorta began with anesthetizing a lab rat and sacrificing it with a spinal fracture. Next, the heart was carefully removed through the following steps: (1) an incision was made from the xiphoid process to the lateral ends of the edges of the ribs, (2) through the ribs along the left and right anterior axillary lines to provide a costal thoracotomy, and (3) the chest was deviated upward. These steps provided full access to the heart. Sections of the vena cava and aorta completed the procedure for extracting the heart, after which the organ was washed with Tyrode’s salt solution (Sigma-Aldrich Co.) with heparin and transferred to a Petri dish with the same solution for further manipulations.

The process of attaching the heart to a cannula (a needle with a soft polymer sheath) through the aorta was performed with several turns of surgical thread. From the moment the heart was removed from the body until the start of perfusion, no more than 10 minutes passed through the cannula.

According to Langendorff, perfusion of the heart was performed using a special installation. The setup consisted of two main parts: a perfusion circuit and a recording optical system based on a high-speed imaging setup (Olympus MVX-10 Macro-View fluorescent microscope (Olympus Co., Tokyo, Japan) equipped with a high-speed Andor iXon-3 Camera 897-U (Andor Technology Ltd., Belfast, UK)). The perfusion circuit was prepared for constant circulation (Masterflex L/S Digital Drive, 600 rpm; 115/230 VAC, Masterflex L/S Easy-Load® II Pump Head, SS Rotor; 2-Channel (Cole-Parmer Instrument Company, Vernon Hills, IL, USA)) of a fixed volume of fluid, maintaining the temperature of the perfusate throughout the system (Cole-Parmer Polystat Standard 6.5 L Heated Bath, 150 C, 115 V AC/60 Hz (Cole-Parmer Instrument Company, Vernon Hills, IL, USA)), including the heart chamber, and oxygenating the solution (Cole-Parmer Bubble trap, Water Jacketed Reservoir, Oxygenating Bubbler (Cole-Parmer Instrument Company, Vernon Hills, IL, USA)). The total volume of fluid circulating in the unit was optimized using a compact PDMS polymer heart chamber. The minimum volume that allowed the heart to be perfused with an oxygenated, heated solution was reduced to 30 mL.

### 4.11. Optical mapping protocols

For cell culture, optical mapping with Fluo4-AM (Lumiprobe, 1892−500ug) and Di-8-ANEPPS (Thermo Fisher Scientific, D3167) was performed in Tyrode’s salt solution (pH 7.25–7.4) according to the protocol previously described in [65]. The signal was recorded with a 128 × 128 pixel resolution and a sampling frequency of 130 frames per second (Olympus MVX-10 Macro-View fluorescent microscope (Olympus Co., Tokyo, Japan) equipped with high-speed Andor iXon-3 EMCCD Camera (Andor Technology Ltd., Belfast, UK)).

In general, the mapping protocol for the whole heart was similar to the protocol for cells [66]. Optical mapping with Fluo4-AM (Lumiprobe, 1892−500ug) and Di-8-ANEPPS (Thermo Fisher Scientific, D3167) was also performed in Tyrode’s salt solution (pH 7.25 to 7.4). The signal was recorded at a resolution of 128 × 128 pixels and a sampling frequency of 130 frames per second using the same settings. The only difference in optical mapping was that the heartbeats were removed by BDM (Sigma), adding to the solution in concentration 10 mM when the heart was stained for fluorescence Fluo4-AM (Lumiprobe, 1892−500ug) in concentration 2.8 ug/ml and Di-8-ANEPPS (Thermo Fisher Scientific, D3167) in concentration 15 ug/ml.

All experiments were performed at 37°C during the observations. The experiments used electrode stimulation. The electrical stimulation duration was set at 20 ms, with the amplitude adjusted within the range of 5–8 V depending on the tissue culture or the excitation threshold of the heart. The stimulation interval varied from 200 to 1000 ms. The stimulus was set using a generator (2MHz USB PC Function Generator, PCGU100 (Velleman, Gavere, Belgium)). Platinum electrodes were used in the work.

Data processing was fulfilled using the ImageJ program and the associated plugins (http://rsbweb.nih.gov/ij/ (accessed on 5 May 2023)). ImageJ plugin (time-lapse color-coder) was used to build pseudo-3D images and activation maps. Principal analysis was performed in Wolfram Mathematica 12.

### 4.12. Data processing and statistical analysis

Preliminary processing of optical mapping data was performed using ImageJ (v1.54p). To construct activation maps, background subtraction was applied to the videos, followed by the Kalman filtering algorithm for noise removal and Gaussian blurring. The activation map generation algorithm was implemented in Python (v3.11.13) using the following libraries: Matplotlib (v3.10.0), NumPy (v2.0.2), SciPy (v1.15.3), and Pandas (v2.2.2). The algorithm is based on increasing the luminescence intensity of each individual pixel by a certain percentage relative to its average intensity. This approach enhances the method’s sensitivity compared to the standard technique of applying a uniform absolute threshold to all pixels.

For analyzing calcium wave propagation, action potential propagation, and NADH photobleaching, plots were generated with preliminary processing in ImageJ (by the Kalman filtering algorithm for noise reduction), followed by data processing in Microsoft Excel and further plot generation and statistical analysis in Python (v3.11.13) with the same libraries. Statistical analysis was performed using the Anderson–Darling test with a 5% significance level.

For the analysis of α-actinin expression percentage in cells, photographs of cells after immunofluorescent staining were taken. Based on the expression data in the photographs, the percentage of area occupied by cells expressing alpha-actinin was calculated in relation to the area occupied by all cells (f-actin) using ImageJ standard functions.

## 5. Conclusions

Electromechanical wave propagation is essential for cardiac function; its disruption by fibrotic scars after myocardial infarction causes arrhythmias. This study develops minimized chemical cocktails for direct fibroblast-to-cardiomyocyte transdifferentiation (TdCM). Optimized 4-component protocols generated functional induced cardiomyocytes (iCMs) from rat and human fibroblasts, with 56–83% cells expressing α-actinin. Electrophysiological profiling confirmed the acquisition of cardiomyocyte-like electrical properties: human iCMs exhibited action potentials (−80 mV resting potential) and robust sodium currents (∼4000 pA). Crucially, partial reprogramming was sufficient to establish conduction pathways that exceeded the percolation threshold (20–30%), enabling electrical propagation. *In vivo* testing showed no hematological toxicity, while optical mapping revealed 84% conductive area in treated infarcts (vs. 71% controls). Our findings demonstrate that minimized cocktails restore scar conductivity without complete cellular conversion, advancing translatable regenerative strategies. Taken together, our findings demonstrate that minimized cocktails can establish partial electrical continuity in the scar by restoring conductivity to a level near the percolation threshold. This outcome is achieved without complete cellular conversion or full structural/contractile maturation, highlighting a translatable regenerative strategy targeted primarily at electrical conduction repair.

## Abbreviations

CVDs: Cardiovascular Diseases
MI: Myocardial Infarction
IND: Ischemic Heart Disease
CFs: Cardiac Fibroblasts
ECM: Extracellular Matrix
iPSCs: Induced Pluripotent Stem Cells
MSCs: Mesenchymal Stem Cells
iCMs: Induced Cardiomyocyte-like Cells
REF: Rat Embryonic Fibroblasts
RNF: Rat Neonatal Fibroblasts
HAF: Human Atrial Fibroblasts
TdCM: Fibroblast-to-cardiomyocyte Transdifferentiation
iPSC-CMs: iPSC-derived cardiomyocyte
